# Seismic Performance of Precast Reinforced Concrete Beam–Column Connections with Embedded Steel Sections

**DOI:** 10.3390/ma19061233

**Published:** 2026-03-20

**Authors:** Banu Ardi Hidayat, Yanuar Haryanto, Hsuan-Teh Hu, Feng-Chien Su, Fu-Pei Hsiao, Laurencius Nugroho, Bobby Rio Indriyantho, Erich

**Affiliations:** 1 Department of Civil Engineering, National Cheng Kung University, Tainan 701, Taiwan; 2 Department of Civil Engineering, Universitas Diponegoro, Semarang 50275, Indonesia; 3 Department of Civil Engineering, Universitas Jenderal Soedirman, Purwokerto 53122, Indonesia; 4 National Center for Research on Earthquake Engineering, Taipei 106, Taiwan; 5 Feng Chien Professional Civil Engineer Workshop, Tainan 701, Taiwan

**Keywords:** precast reinforced concrete, beam–column connection, embedded steel section, cyclic loading, seismic performance

## Abstract

Precast reinforced concrete (RC) structures offer advantages in terms of construction efficiency and quality control; however, their seismic performance is governed by the behavior of the beam–column connections. This study presents an experimental investigation of the cyclic response of precast RC beam–column joints that include a composite steel connection, designed to enhance strength, stiffness, and damage control in critical regions. A composite joint specimen was tested under displacement-controlled cyclic loading, and its behavior was compared with that of a corresponding pure RC connection. Experimental results showed that the composite configuration effectively prevented premature failure at the beam–column interface, relocated plastic hinges away from the joint core, and significantly improved the load-carrying capacity, stiffness, and energy dissipation. To interpret the experimental observations and examine the internal stress transfer and evolution of damage, a three-dimensional nonlinear finite-element model was developed. The simulations reproduced the observed modes of failure, shapes of deformation, hysteretic responses, and moment distribution trends, particularly in the post-yield and strain-hardening ranges. Although the pinching effects observed experimentally were not fully captured numerically, the overall levels of agreement in the ultimate strength and plastic hinge locations were satisfactory. The combined results indicate that composite steel-reinforced precast beam–column joints represent a promising solution for improving seismic performance.

## 1. Introduction

Precast reinforced concrete (RC) structures have gained prominence in civil engineering due to their benefits in terms of construction efficiency, quality assurance, improved project scheduling, and reduced on-site labor [1,2,3,4]. The manufacture of structural components in controlled industrial environments can ensure consistent material properties and precise dimensions before on-site assembly. Consequently, precast systems have emerged as an efficient solution for modern residential, commercial, and industrial building construction, including those situated in seismic zones [5,6,7].

In precast RC frame systems, beam–column joints are an important aspect when determining structural performance. Previous studies have demonstrated that these joints experience significant shear stress, combined bending and axial forces, and repetitive cyclic deformation during seismic events [8,9,10,11]. Investigations into monolithic RC frames and traditional precast connections have revealed that insufficient joint detailing can result in premature damage concentration, loss of stiffness, and brittle failure mechanisms localized inside the joint core [12,13]. Such failures may substantially diminish the energy dissipation capacity and affect the seismic performance and integrity of the overall structure [14].

Enhancing the seismic performance of the joint region in the precast concrete frame joints is therefore critically important [15,16,17]. To address this issue, numerous beam–column connection methods have been proposed to enhance joint performance, including improved reinforcement details, mechanical connectors, and steel-supported or composite connection systems [18,19,20,21]. Although these methods can improve the strength and confinement, many existing solutions depend on external steel components, complex mechanical anchorage systems, or conventional steel-reinforced concrete (SRC) concepts that do not specifically address the problem of plastic hinge relocation mechanisms in precast connections. There has been little research into the effectiveness of internally embedded steel connection elements that are designed to shift inelastic demand from the joint core to the adjacent beam region under displacement-controlled cyclic loads.

The design of the composite steel connection detail presented in this paper is aligned with the seismic capacity design principles established in structural design standards, such as ACI 318 [22], ACI CODE-550.3-13 [23], and EN 1998-1 [24], which emphasize strong column–weak beam behavior and protection of the joint core region in moment-resisting frames. The aim of the approach introduced here is to improve the implementation of established code principles in precast systems by incorporating an internally embedded steel element that reinforces the joint interface and intentionally promotes the formation of plastic hinges in the adjacent beam region. This technique aligns with hierarchical strength requirements and aims to guarantee that inelastic deformation occurs in ductile beam segments instead of the joint core, therefore supporting established seismic performance objectives for RC frame systems.

Furthermore, although previous studies have reported strength augmentation in steel-assisted joints [25,26], a comprehensive evaluation of the failure mechanisms, plastic hinge relocation behavior, and cyclic degradation characteristics of precast composite steel-reinforced concrete joints is required. The inconsistencies between experimental data and numerical predictions, especially with regard to stiffness degradation and pinching effects, have not been adequately examined [27,28]. A comprehensive understanding of the impact of an embedded composite steel connection on stress transfer and damage is therefore essential.

In particular, there is a lack of extensive experimental data about the cyclic behavior, causes of failure, and plastic hinge formation in these composite precast joints under displacement-controlled stress that simulates seismic events [25,29]. Furthermore, although nonlinear finite element (FE) analysis has been extensively used to examine RC joints, inconsistencies between numerical predictions and experimental findings remain, especially with regard to accurately representing cyclic degradation, pinching behavior, and damage localization in precast connection systems. These constraints underscore the necessity for integrated experimental and numerical investigations to deepen the current understanding of the structural behavior of composite precast beam–column joints.

This paper fills this gap by describing an integrated experimental test and computational FE analysis of precast RC beam–column joints, including a composite steel connection. An experimental program was carried out involving a composite-reinforced joint specimen and an equivalent pure RC control specimen under monotonic and cyclic loading conditions. The assessments were formulated to assess the load–displacement response, stiffness deterioration, energy dissipation, mechanisms of failure, and repositioning of plastic hinges. A three-dimensional (3D) nonlinear FE model was developed simultaneously to replicate the experimental configuration and investigate the internal stress transfer, damage progression, and interaction between the concrete and steel components. This research work combined experimental observations with numerical FE analysis to elucidate the effectiveness of composite steel-reinforced connections in enhancing joint performance and regulating damage distribution. The results enhance the comprehension of damage control and force transfer mechanisms in precast frame systems and facilitate the formulation of more reliable design and evaluation strategies for precast RC structures in seismically active areas.

## 2. Experimental Program

### 2.1. Materials

The material properties of precast concrete were ascertained through compressive strength tests of standard cylindrical samples with a diameter of 150 mm and a height of 300 mm. The compressive tests were performed at National Cheng Kung University (NCKU), Taiwan, in accordance with ASTM C39/C39M-21 [30], using a 100-ton universal testing machine (UTM) manufactured by Shimadzu Corporation, Tokyo, Japan. The experimental results served as a reference for establishing the material parameters adopted in both the experimental analysis and numerical simulation. A total of 12 specimens were evaluated, and the complete stress–strain response was documented throughout the testing process. The average compressive strength (*f_c_^′^*) was found to be 41.22 MPa, with the corresponding ultimate compressive strain measured as 0.53%. The modulus of elasticity (*E*) was calculated in accordance with ACI 318 [22] based on the average measured compressive strength, resulting in a value of 30,174.94 MPa. Poisson’s ratio for precast concrete was assumed to be 0.3, a value that is commonly adopted for concrete with normal strength. While the compressive properties were derived directly from experimental observations, the tensile properties were not independently measured. The tensile strength *f_t_* was established using the empirical formula recommended in the ACI guidelines [22], as shown in Equation (1). This method is frequently used for normal-strength concrete in the absence of direct tensile testing. Table 1 summarizes the measured value for the compressive strength and the other parameters derived from the experimental tests.

(1) $$f_{t}=0.56\sqrt{{f_{c}}^{\prime }}$$

Various steel materials were used in the composite steel connection and reinforcing bars. The configuration of the reinforcement for the RC beam–column joint specimens included three distinct bar sizes: #4 (D13) for the stirrup reinforcements of both the column and beam, #7 (D22) for the longitudinal bars of the beam, and #10 (D32) for the longitudinal reinforcements of the column. Moreover, a steel profile of the H-304×301×11×17 type was adopted for the I-shaped steel structure implemented in the composite joint specimen. Uniaxial tensile tests were performed at NCKU, Taiwan, using a UTM with a capacity of 50 tons provided by Shimadzu Corporation, Tokyo, Japan, to ascertain their actual tensile properties, in compliance with ASTM A370-18 [31]. In each test, a constant-rate loading protocol was maintained until fracture occurred in the bar samples.

The I-shaped steel connection was found to have a yield strength of 247.13 MPa. The longitudinal reinforcement bars had yield strength values of 411.88 MPa and 448.61 MPa, respectively, for the #7 and #10 bars, whereas the stirrup reinforcement had a yield strength of 274.59 MPa. These values were established according to the material specifications supplied for the experimental specimens. All steel materials were assumed to have an elastic modulus of 200,000 MPa, a Poisson’s ratio of 0.3, and a density of 7800 kg/m^3^.

### 2.2. Test Specimens

The structural performance of precast concrete structures strongly depends on the connections between the beam and column members. In this study, a composite steel-reinforced connection was employed to guarantee sufficient stability and load transfer. This connection included a structural steel component within the beam–column joint area, thereby creating a composite system that was designed to improve the moment resistance, stiffness, and damage mitigation while minimizing premature failure in the joint core.

Two beam–column joint specimens were designed and manufactured by Ming Rong Yuan Business Co., Ltd., Pingtung, Taiwan, comprising a composite-reinforced joint (CRJ) specimen and a pure reinforced joint (PRJ) specimen that was used as a control. The composite-reinforced specimen represented the principal design approach examined in this study, whereas the pure reinforced specimen consisted of a conventional monolithic beam–column connection without embedded steel components. The experimental program was intentionally designed in the form of controlled comparative research, in which both specimens had a similar shape, reinforcing configuration, concrete strength, and axial load magnitude and were subjected to the same loading technique, with the connection configuration as the only variable. This method facilitated a direct assessment of the impact of the composite steel connection on the load–displacement response, failure mechanism, plastic hinge development, and damage progression. It is important to note that only a single specimen was evaluated for each setup, meaning that the findings can be regarded as mechanistic comparisons rather than statistically generalized conclusions. Although this restricted specimen count precludes an evaluation of variability, the experimental results were validated by comprehensive FE simulations to enhance the reliability of the observed behavioral trends.

**Figure 1:**
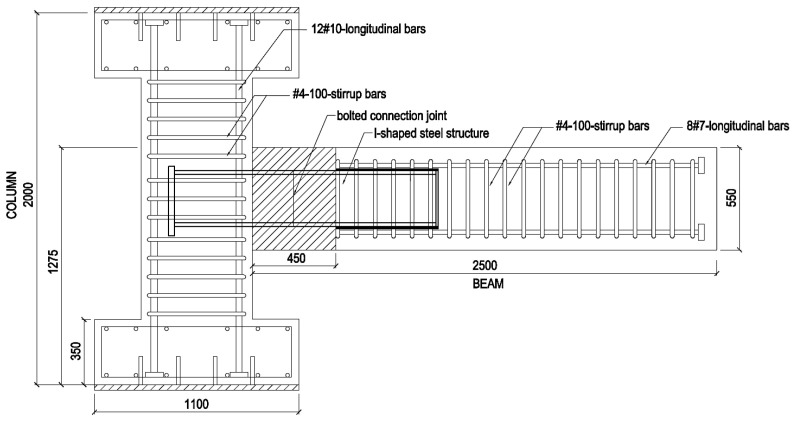
Schematic diagram of the composite-reinforced joint (CRJ) specimen (dimensions in mm).

The CRJ specimen consisted of three primary components: an RC column, an RC beam, and an I-shaped steel section that functioned as the connecting element. The column had a cross-section of 600 mm × 600 mm and a height of 2000 mm, whereas the beam section measured 450 mm × 550 mm with an overall length of 2500 mm. The steel connection element consisted of an I-shaped steel section with a depth of 304 mm, flange width of 301 mm, web thickness of 11 mm, and flange thickness of 17 mm (304 mm × 301 mm × 11 mm × 17 mm), with a total length of 1450 mm. The column was strengthened with 12 #10 longitudinal bars and confined using #4 stirrups at intervals of 100 mm, while the beam section was reinforced with eight longitudinal #7 bars and transverse reinforcement consisting of single-leg #4 stirrups spaced at intervals of 100 mm. The specimen was anchored with a hinge pin at the column base and a roller support at the free end of the beam to replicate the experimental boundary conditions. The geometry of the specimen and the configuration of the reinforcement are illustrated in Figure 1.

Before selecting the embedded steel section used for the CRJ specimen, several steel configurations were evaluated at the preliminary stage of numerical design. Two composite configurations were examined to assess their structural behavior and feasibility. The first configuration consisted of a larger I-shaped steel section of size 660 mm × 500 mm × 16 mm × 32 mm with a length of 2000 mm. This design had a significantly higher maximum load capacity and stiffness, but the numerical findings revealed excessive damage to both the beam and column regions, which violated the strong column–weak beam principle and exceeded the capacity of the available testing facilities. A second configuration was based on a smaller I-shaped steel section (304 mm × 301 mm × 11 mm × 17 mm) measuring 1400 mm in length. With this design, the laboratory constraints were effectively met, and column damage was minimized; however, premature plastic hinge formation was still observed near the beam–column connection, with localized steel buckling. Based on these assessments, the final CRJ configuration was optimized to achieve a beam-controlled response while satisfying the structural design criteria and experimental feasibility requirements.

**Figure 2:**
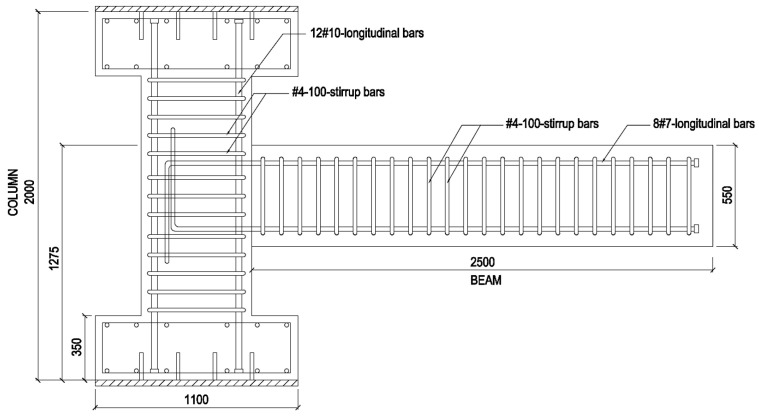
Schematic diagram of the pure reinforced joint (PRJ) specimen (dimensions in mm).

The PRJ specimen was constructed as a control specimen representing a conventional RC connection, comprising an RC column and a beam connected via a conventional monolithic joint construction, as seen in Figure 2. The geometric measurements were identical to those of the composite-reinforced specimen except for the absence of the I-shaped steel connection. The reinforcement configuration of the pure reinforced specimen mirrored that of the composite specimen, with 12 #10 longitudinal bars and #4 stirrups spaced at 100 mm in the column and eight #7 longitudinal bars with single-leg #4 stirrups with spacings of 100 mm in the beam. The column had a cross-section of 600 mm × 600 mm and a height of 2000 mm, while the beam section measured 450 mm × 550 mm and had an overall length of 2500 mm. The boundary conditions for the pure reinforced specimen were equal to those of the composite-reinforced specimen, allowing for a consistent experimental comparison. The geometry and reinforcing specifications of both specimens are summarized in Table 2.

### 2.3. Loading Configuration and Experimental Setup

**Figure 3:**
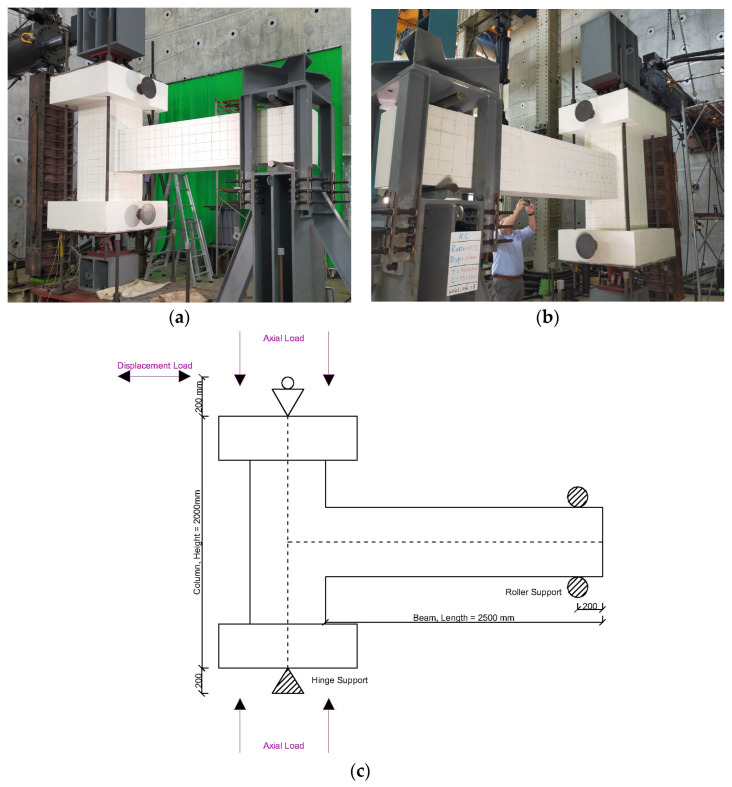
Experimental setup adopted for beam–column joint testing: (**a**) photograph showing the test of the CRJ specimen; (**b**) photograph showing the test of the PRJ specimen; (**c**) schematic diagram of the load design.

The loading configuration and experimental setup adopted in this study were designed to reproduce the structural response of the specimens subjected to monotonic and cyclic lateral loading, as depicted in Figure 3. The test arrangement consisted of an RC column linked to a cantilever beam, representing a standard sub-assembly extracted from a moment-resisting frame. This configuration facilitated direct observation of the joint behavior, force transfer mechanisms, and development of plastic hinges under lateral deformation. A gravitational force representing the self-weight effects, axial load, and lateral load was imposed on the beam–column joints, and a continuous axial load of 200 tons was applied at the column ends during the test to simulate sustained axial force conditions. Based on a gross cross-sectional area for the column of 0.36 m^2^ and a measured value for *f_c_^’^* of 41.22 MPa, the applied axial force corresponded to an axial load ratio of approximately 0.13. This value is consistent with the typical axial load ratios found in RC moment-resisting frame columns under seismic loading, thereby facilitating an accurate simulation of gravity load conditions in prototype structures. Lateral loading was exerted at the top of the column by displacement control to generate simultaneous bending and shear forces in the region of the beam–column joint. The vertical distance between the lower and upper supports was 2400 mm, with each support segment measuring 200 mm in height.

The experimental loading program included both monotonic and cyclic loading scenarios. Monotonic loading was initially implemented under displacement control to allow for an examination of the fundamental nonlinear response of the specimens. A maximum lateral displacement of 150 mm was applied at the top of the column for both the composite-reinforced and pure-reinforced specimens. Following the monotonic tests, cyclic loading was implemented to replicate the seismic loading conditions using a servo-controlled hydraulic actuator to facilitate the gradual accumulation of damage and degradation processes typical of seismic loading. The specimens were subjected to displacement-controlled cyclic loading at a low rate of roughly 0.5 mm per second; at this loading rate, inertia effects were considered insignificant, meaning that the response could be regarded as quasi-static.

Although a separate loading-rate sensitivity analysis was not performed, the chosen displacement-controlled rate of 0.5 mm/s is aligned with the gradual quasi-static condition commonly used in laboratory cycle testing [12,32]. No oscillatory behavior or dynamic amplification was observed in the reaction force histories, and the numerical analysis was conducted without artificial damping, confirming that inertia effects were minimal. It could therefore be concluded that the stiffness degradation and energy dissipation were predominantly influenced by material nonlinearity rather than loading-rate effects.

**Figure 4:**
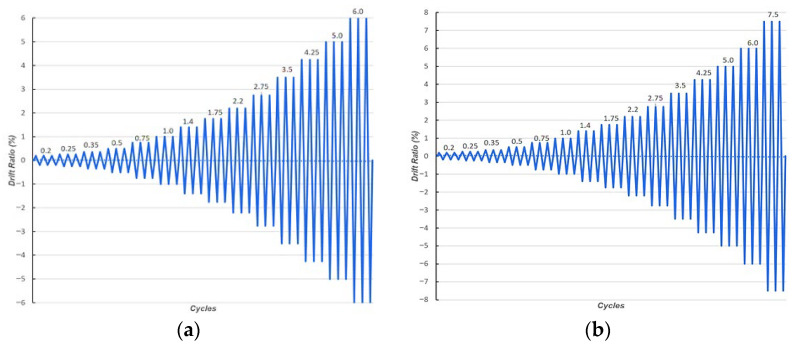
Displacement loading cycles for the beam–column joint specimens: (**a**) CRJ specimen; (**b**) PRJ specimen.

The cyclic displacement history followed the loading protocol specified in ACI T1.1R-01 [33]. The lateral drift ratio for the composite-reinforced specimens was incrementally raised to 6% before failure, whereas for the pure reinforced specimens, it was elevated to 7.5%, as illustrated in Figure 4. The composite-reinforced specimen underwent 42 loading cycles, while 45 loading cycles were used for the pure reinforced specimen. The loading specifications, laboratory testing setup, and drift limits were later incorporated into the numerical simulations to maintain consistency between the experimental and numerical investigations.

Instrumentation was set up to assess both the global and local structural responses. Lateral displacements were recorded using linear variable differential transformers positioned at the top of the column and along the beam to assess the deformation profiles. The applied load was quantified with a calibrated load cell incorporated with the actuator. Strain gauges were affixed to the longitudinal reinforcement bars in the beam and the column adjacent to the joint location to monitor the yielding behavior and strain distribution. Additional strain gauges were affixed to the steel connection element in the composite-reinforced specimens to observe the stress development within the steel component.

## 3. Finite Element Modeling

A nonlinear FE analysis was performed to model the structural response of the precast RC beam–column joint specimens subjected to monotonic and cyclic loads. This analysis included material nonlinearity via constitutive models for concrete, reinforcing steel, and the composite steel connection, and geometric nonlinearity was included to handle the substantial displacement effects at elevated drift levels. The FE modeling framework, encompassing the specimen geometry, loading protocol, material representation, and boundary conditions, was established to precisely replicate the experimental test configuration and facilitate a dependable comparison of the load–displacement response, modes of failure, and plastic hinge formation.

### 3.1. Element Identification and Model Geometry

The selection of suitable FE types was essential in order to model the nonlinear behavior of steel and RC elements effectively. ABAQUS software (ver. 2017) offers 3D eight-node solid elements in several formulations, including fully integrated, limited integration, hybrid, and incompatible mode versions. Each formulation has distinct numerical attributes that may affect the convergence, accuracy, and damage representation, especially when nonlinear material models are included.

A preliminary numerical evaluation was performed using a simplified cantilever column model exposed to lateral displacement in order to determine the appropriateness of various 3D eight-node solid (C3D8) element formulations in conjunction with the concrete damage plasticity (CDP) model. The comparison included incompatible mode (C3D8I), fully integrated (C3D8), reduced-integration (C3D8R), hybrid (C3D8H) formulations, and their respective variations. The assessment criteria were predicated on the maximum tensile and compressive damage parameters, as summarized in Table 3.

Several formulations, such as C3D8I, C3D8, C3D8H, and C3D8IH, yielded damage levels surpassing 100%, signifying the accumulation of nonphysical damage and numerical instability. These formulations also had problems in converging under nonlinear loading conditions. The reduced C3D8R produced a maximum tensile damage of 90.96% and compressive damage of 12.06%, both of which were within acceptable physical limits, while ensuring steady convergence. Comparable stability was noted for C3D8S and C3D8RH; however, C3D8R yielded the most computationally efficient solution without artificial damage amplification.

**Figure 5:**
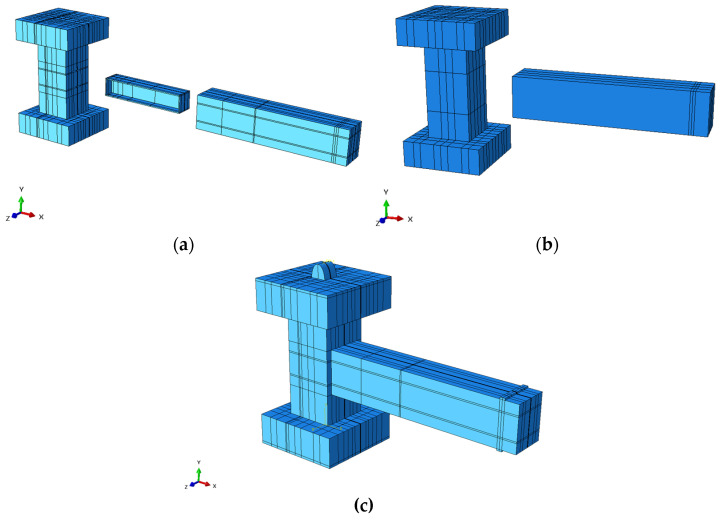
Details of the solid element in the FE model: (**a**) CRJ specimen; (**b**) PRJ specimen (both with the separated elements of the RC column, RC beam, and I-shaped steel); (**c**) comprehensive assembly model of the beam–column joint specimen.

Based on these quantitative comparisons, the C3D8R elements were chosen to simulate the RC sections of the beam and column. C3D8I elements were used for the structural steel components in the composite connection, since this formulation was more appropriate for ductile materials and bending-dominated behavior, while reducing the effects of shear locking on the element. All reinforcing bars were represented using 3D two-node truss elements (T3D2), as this efficiently facilitated the transfer of axial force, while insignificant bending and shear effects relevant to the embedded reinforcement in the parent concrete were neglected. Figure 5a illustrates the solid element employed in the numerical model, with separate column, beam, and I-shaped steel parts. In the PRJ specimen model, there were only two solid elements, namely the column and beam components, as seen in Figure 5b. Subsequently, all components were assembled into an integrated beam–column structure, as depicted in Figure 5c.

### 3.2. Mesh Strategy

**Figure 6:**
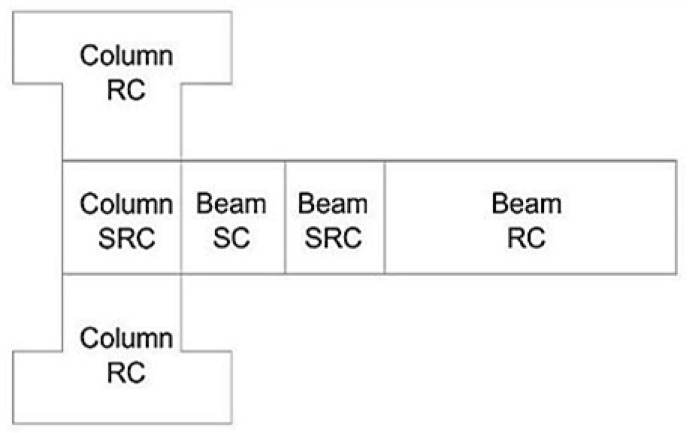
Parts of the numerical model. The labels RC, SRC, and SC represent the reinforced concrete, steel-reinforced concrete, and steel–concrete regions of the beam–column joint structure, respectively.

To precisely capture the structural response of the composite and pure reinforced beam–column joint specimens while ensuring computational efficiency, various mesh densities were used for the different sections of the numerical model. The model was categorized into five sections according to the structural configuration and material composition: (i) the RC column region, (ii) the SRC column region, (iii) the steel–concrete (SC) beam region, (iv) the SRC beam region, and (v) the RC beam region, both with and without embedded steel elements, as depicted in Figure 6.

A more refined mesh discretization was applied in areas that were expected to experience significant stress concentrations, damage progression, and plastic hinge development. The steel–concrete and steel-reinforced concrete sections were discretized with an element size of 100 mm. These regions included the beam–column connection area, which was characterized by the composite interaction between the embedded steel element and the surrounding concrete, meaning that a higher resolution was required to precisely capture the nonlinear behavior. The steel–concrete region of the beam was specifically designated as the beam–column connection area, with the design goal of preventing failure in this region and facilitating plastic hinge formation in the neighboring SRC regions of the beam.

A coarser mesh discretization was adopted for the RC column and beam areas farther from the joint, where lower stress gradients were expected. This method facilitated a decrease in the total number of elements while maintaining the precision of the overall structural response. The reinforcing steel bars were discretized using truss elements with an element length of 150 mm to ensure compliance with the surrounding concrete mesh and stable force transfer via the embedded constraint.

**Figure 7:**
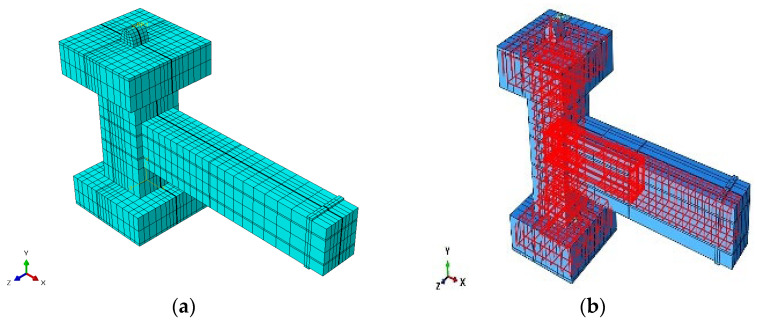
Geometric details of the FE model of the CRJ specimen: (**a**) concrete section; (**b**) reinforcement configuration. Color variations indicate the different elements applied in the model, with cyan representing the concrete parts and red indicating the steel and reinforcing bars embedded inside the blue-colored concrete. The black lines depict the FE mesh used for structural discretization.

**Figure 8:**
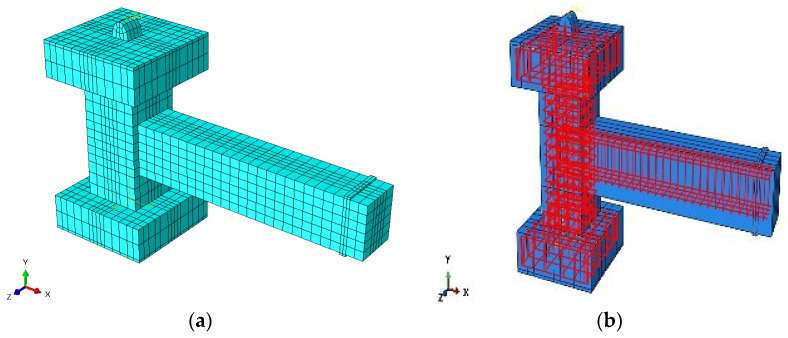
Geometric details of the FE model of the PRJ specimen: (**a**) concrete section; (**b**) reinforcement configuration. Color variations denote the distinct elements used in the model, with cyan signifying the concrete components and red representing the steel and reinforcing bars placed within the blue-colored concrete. The black lines show the finite element mesh employed for structural discretization.

The mesh structure implemented here achieved a compromise between numerical precision and computing efficiency: a finer mesh throughout the entire structure would have improved the local accuracy but would have considerably raised the computational time and memory requirements. Consequently, the mesh refinement focused on crucial areas influencing the structural behavior and was considered suitable for accurately capturing the response of the beam–column joint specimens examined in this investigation. Figure 7 and Figure 8 depict the comprehensive FE mesh discretization and the overall geometry of both the composite and pure reinforced numerical models.

Although a formal multi-level mesh convergence study was not performed, the chosen mesh size was established based on both the numerical stability and the characteristic length requirements of the CDP model. In damage-based constitutive models, excessive mesh refinement can lead to false strain localization and mesh-dependent softening responses. The 100 mm mesh adopted for the SC and SRC regions yielded consistent global load–displacement responses, devoid of spurious damage concentration or unrealistic stiffness degradation. Furthermore, the predicted damage localization and plastic hinge regions were aligned with the experimental findings, indicating that the selected mesh arrangement closely represents the structural behavior of the specimens while ensuring computational efficiency.

### 3.3. Boundary Conditions and Interaction Model

Interaction definitions are essential in FE analysis, as they directly affect the numerical stability, convergence characteristics, and accuracy of the simulated structural response. In this present study, the interaction models were carefully designed to replicate the experimental conditions while avoiding unnecessary constraints that could lead to excessive computational expense or convergence difficulties.

The connection between the beam and column was simulated with a tie constraint, thus ensuring complete displacement compatibility at the interface. This assumption represents a perfectly bonded connection without relative slip and was adopted to mirror the monolithic casting configuration of the experimental specimens. Similarly, the reinforcing steel bars were modeled using the embedded region constraint, which corresponds to ideal bond behavior between the truss elements representing the reinforcement bars and the parent concrete solid elements. In the composite strengthened numerical model, tie restrictions were used to simulate the structural steel connection, effectively connecting the steel section to both the RC beam and column. This approach is based on the assumption of perfect interaction between the steel connection and the adjacent concrete, thus preventing slip or separation during loading. Although this modeling technique improves the numerical stability and computational efficiency of the analysis, it naturally neglects the bond-slip behavior and interface degradation mechanisms that may develop under cyclic loading. As a result, the simulation does not explicitly account for local reinforcement slip, crack-induced interface separation, and bond deterioration effects. This bond assumption should be recognized as a limitation of the model when interpreting the cyclic degradation behavior.

**Figure 9:**
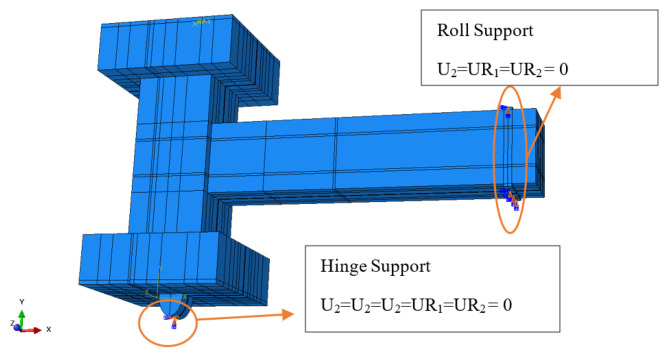
Boundary conditions for the beam–column joint model. The quantities U and UR represent the translation and rotational movement of the model, respectively.

Boundary conditions were established to replicate the experimental support configuration. The base of the column was designed as a hinge support by constraining translational degrees of freedom in all directions while permitting rotational freedom. To illustrate the experimental support behavior established by point supports, rotational constraints were imposed in specific directions to prevent unintended rigid-body rotation. At the beam ends, roller supports were introduced using line constraints to allow for axial translation while limiting vertical movement. Additional rotational constraints were implemented to provide numerical stability and to prevent inaccurate rotational effects linked to the modeling of the support. Figure 9 illustrates the numerical boundary condition configuration of the FE model structure created using ABAQUS software, with direction 1 representing the X-direction, direction 2 denoting the Y-direction, and direction 3 indicating the Z-direction.

### 3.4. Material Constitutive Model

The mechanical behavior of the steel reinforcement bars and structural steel components was modeled using an idealized elastic–perfectly plastic constitutive model to represent the yielding and post-yield behavior of the reinforcement under both monotonic and cyclic loading situations. All steel materials were assumed to have a density of 7800 kg/m^3^, an elastic modulus of 200 GPa, a Poisson’s ratio of 0.30, and strength based on the material specifications of the experimental specimens. An elastic–perfectly plastic model was intentionally chosen to neglect the effects of material strain hardening, as this simplification improved numerical stability and allowed the investigation to focus on the global structural response and mechanisms of plastic hinge development. Although neglecting strain hardening may give slight underestimates of the post-yield stiffness and cumulative energy dissipation of the steel components, the peak load capacity and failure location were primarily influenced by the composite interaction and concrete damage behavior, meaning that the impact of this simplification can be considered limited. Nonetheless, this assumption must be acknowledged as a modeling simplification when analyzing the cyclic degradation and energy dissipation results.

The uniaxial compressive characteristics of concrete were derived based on empirically acquired stress–strain data from conventional compression experiments. The precast concrete material was characterized by an average compressive strength of 41.22 MPa. These data were later used to characterize both the elastic modulus and the nonlinear plasticity behavior of concrete in the numerical model. The modulus of elasticity was determined following ACI 318 [22], yielding a value of 30,174.94 MPa. Poisson’s ratio for concrete was estimated as 0.30, a value within the generally accepted range for normal-strength concrete. The density of precast concrete was assumed to be 2400 kg/m^3^. In addition to the elastic properties, the nonlinear behavior of concrete was characterized by the peak and degradation phases of the compressive response [34], its tensile behavior and post-peak softening phase [35,36,37], and damage-related parameters [38,39], which were calibrated based on the experimental stress–strain curves.

**Figure 10:**
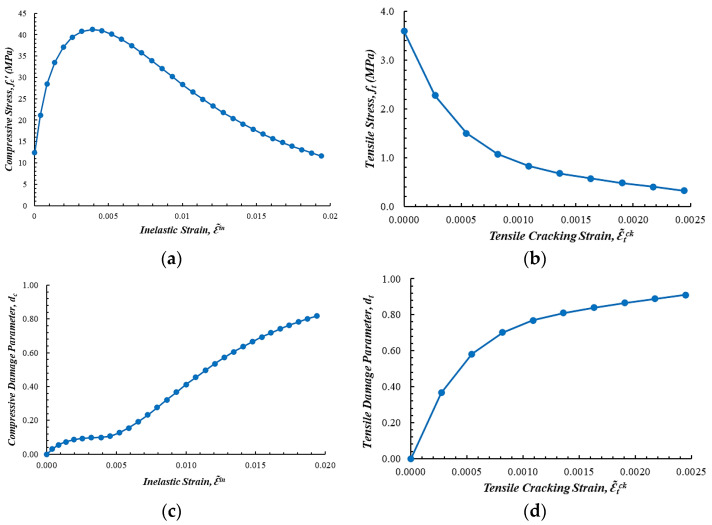
Constitutive material models for concrete elements: (**a**) stress–strain curve under uniaxial compression; (**b**) stress–strain curve under uniaxial tension; (**c**) relationship between the compressive damage parameter and inelastic strain; (**d**) relationship between the tensile damage parameter and inelastic strain.

The nonlinear characteristics of concrete subjected to simultaneous tension and compression were simulated with the CDP model [40,41] provided in ABAQUS. This constitutive model takes into account the irreversible plastic deformation and stiffness deterioration linked to tensile cracking and compressive crushing. Deterioration of the concrete was characterized by two scalar damage factors, related to the tensile and compressive damage, which evolve incrementally when inelastic strains occur. The relationship between compressive stress and strain for the concrete was established using mathematical equations and refined via a trial-and-error method to replicate the experimental results, as shown in Figure 10a. The tensile characteristics of concrete followed the relationship presented in Figure 10b. The corresponding curves for the tensile and compressive damage parameters were derived to illustrate the gradual decrease in stiffness associated with cracking and crushing. Figure 10c,d show the changes in the compressive and tensile damage parameters, representing the percentage of damage to the concrete in relation to its strain behavior.

There are five plasticity parameters that must be specified for this model: the dilation angle (*ψ*), eccentricity (*e*), ratio of biaxial-to-uniaxial compressive strength (*σ*_*b*0_/*σ*_*c*0_), deviatoric surface shape factor (*K_c_*), and viscosity parameter. The default values suggested in the ABAQUS documentation were adopted, with a flow potential eccentricity *e* of 0.1, a ratio of biaxial-to-uniaxial compressive strength *σ*_*b*0_/*σ*_*c*0_ of 1.16, and a deviatoric surface shape factor *K_c_* of 2/3. The dilatation angle *ψ* was established as 35°, a value chosen from the range 30–40° that is often documented for normal-strength concrete materials [42,43]. This value represents a balanced selection that accurately reflects shear dilation behavior without overestimating the strength enhancement generated by confinement. A nonzero viscosity parameter of 0.0005 was introduced to improve the numerical stability in the nonlinear analysis. The viscosity parameter provides viscoplastic regularization that enhances convergence without causing substantial artificial damping when adequately minimized [41]. The chosen value was within the accepted range for cyclic CDP simulations and was confirmed to have a negligible influence on the peak load capacity and failure mechanisms while maintaining consistent convergence during the loading history [12,44,45].

## 4. Results and Discussion

### 4.1. Monotonic Loading Results

Monotonic loading tests were performed to identify the areas of critical damage and stress concentration regions inside the beam–column joint assemblies. In this loading scenario, the distributions of concrete damage and stress development in the steel components were examined, as well as the overall load–displacement response, to provide a clear demonstration of the initial crack behavior, stiffness properties, and failure mechanisms of both specimens.

#### 4.1.1. CRJ Specimen

**Figure 11:**
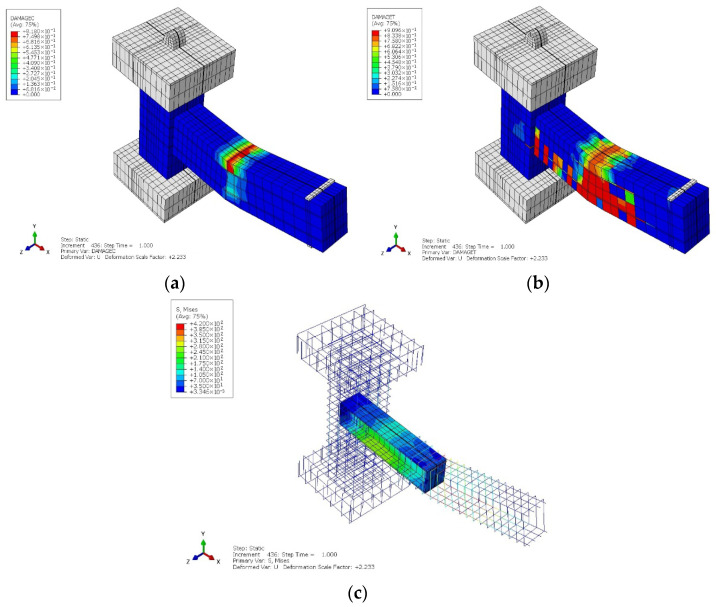
Numerical results for monotonic loading of the CRJ specimen: (**a**) compressive damage; (**b**) tensile damage; (**c**) stress results for the steel component. The color gradient represents the transition from a lower value (in blue color) to a higher value (in red color).

Figure 11a,b present the compressive and tensile damage contours for the CRJ specimen at the end of the monotonic loading phase. The findings demonstrate that damage initiation and crack propagation were primarily concentrated in the SRC-RC area of the beam, whereas the core of the beam–column connection remained predominantly undamaged. This response indicates that the composite steel connection successfully modified the stress transfer mechanism and prevented premature failure within the joint region. The tensile damage pattern illustrated in Figure 11b shows progressive cracking along the length of the beam rather than localized cracking at the joint interface, meaning that the composite-reinforced design effectively facilitates a beam-controlled response under monotonic loading. The relocation of damage away from the joint core corresponds with the intended objective of preventing premature joint failure [46,47,48].

The peak values of the CDP damage variables were analyzed at the end of monotonic loading to quantify the observed damage localization. The maximum tensile damage (DAMAGET) value in the SRC-RC region of the beam approached 0.91, whereas the corresponding values in the beam–column joint core remained below 0.15. Similarly, the value for the compressive damage (DAMAGEC) in the beam region was above 0.81, although the joint core exhibited no compressive degradation. This indicates that the intensity of damage in the SRC-RC region of the beam was approximately six times greater than at the joint interface. These quantitative observations confirm that deterioration was effectively relocated away from the joint core and reveal a beam-controlled failure mechanism under monotonic loading.

With the increase in lateral displacement, a localized stress concentration emerged in the embedded structural steel component, as illustrated in Figure 11c. The interaction between the steel connection and the parent concrete caused increased stresses in the adjacent SRC-RC region of the beam, resulting in yielding of the longitudinal reinforcement and subsequent damage accumulation. The damage pattern observed here verifies that the composite design successfully shifted the plastic hinge from the beam–column connection to the beam region, as intended in the design concept. The interaction between the steel component and adjacent concrete led to an increased stress concentration in the beam area, thus determining the failure mechanism under monotonic loading, which was influenced by beam-controlled flexural-shear behavior rather than joint-controlled failure.

#### 4.1.2. PRJ Specimen

**Figure 12:**
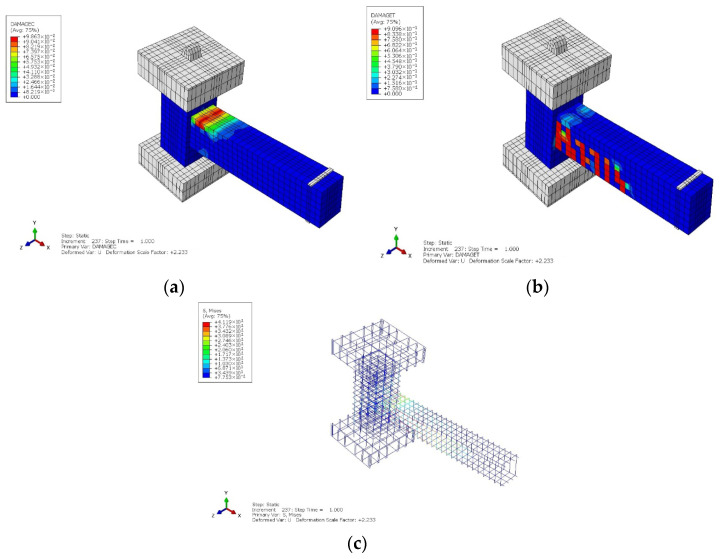
Numerical results for monotonic loading of the PRJ specimen: (**a**) compressive damage; (**b**) tensile damage; (**c**) stress results for the steel component. The color gradient represents the transition from a lower value (in blue color) to a higher value (in red color).

Figure 12a,b show the compressive and tensile damage contours for the PRJ specimen subjected to monotonic loading. Unlike the CRJ specimen, significant damage was predominantly concentrated in the beam–column connection area and the SC zone of the beam. Both compressive crushing and tensile cracking rapidly localized at the joint core, indicating high stress requirements in this crucial area; this was due to the absence of a composite steel connection, which resulted in premature degradation. The pattern of tensile damage reveals a restricted crack distribution along the beam, with damage primarily localized at the connection interface. This behavior reflects a joint-controlled failure mechanism, which is commonly linked to reduced ductility and poor seismic performance. Figure 12c depicts the stress distribution within the steel reinforcement components of the PRJ specimen. In contrast to the composite specimen, the stress contribution of the steel reinforcement is more localized and is focused at the beam–column interface. The absence of a composite steel connection restricts the joint’s capacity to redistribute internal forces, leading to increased stress concentration in the joint area and accelerated damage progression.

The observed damage localization in the PRJ specimen is aligned with the experimental results reported in recent studies of monolithic and precast RC beam–column joints. Prior research indicates that joints without additional confinement or steel reinforcement mechanisms are prone to concentrated diagonal cracking and compressive crushing in the joint core, with rapid deterioration in stiffness under cyclic loading [49,50,51,52,53]. In such cases, the transfer of internal forces towards the beam region is limited, leading to joint-controlled failure mechanisms. The numerical and experimental damage contours identified in this study correspond closely to these recognized behavioral characteristics.

#### 4.1.3. Comparison of CRJ and PRJ Specimens

**Figure 13:**
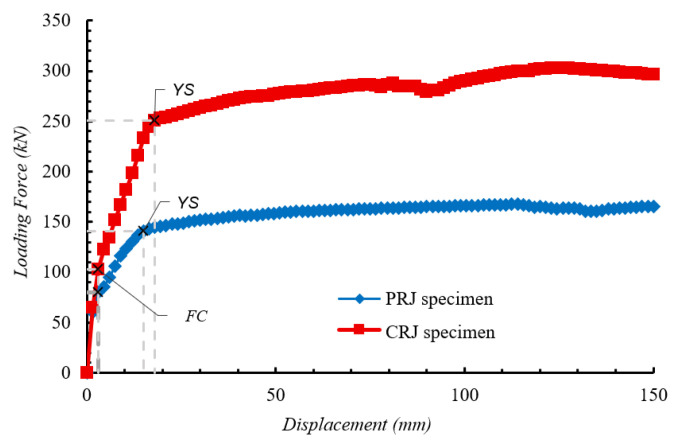
Load–displacement response of PRJ and CRJ specimens for the monotonic loading protocols (*FC* symbolizes the onset of cracking, and *YS* denotes yielding of the longitudinal steel reinforcement, and “x” markers indicate key reference points corresponding to these events).

A direct comparison of the monotonic responses of the CRJ and PRJ specimens underscores the substantial impact of the composite steel connection on the structural performance. From the load–displacement response in Figure 13, it can be seen that both specimens exhibited elastic behavior up to approximately 3 mm of lateral displacement. Beyond this point, cracking was initiated, and the response transitioned into the nonlinear range. For the CRJ specimen, the steel reinforcement yielded after around 18 mm of displacement, subsequently exhibiting a strain-hardening response characterized by continued deformation with a diminished rate of force increase. In contrast, the PRJ specimen showed a more rapid degradation in stiffness after crack initiation, with the reinforcing steel yielding at a lower displacement level of approximately 15 mm. The post-yield response of the PRJ specimen demonstrated minimal strain-hardening capability, resulting in a lower peak force and reduced deformation capacity in comparison to the CRJ specimen. The formation of plastic hinges was localized in the beam–column connection region, indicating a joint-controlled failure mechanism under monotonic loading.

The quantitative comparison presented in Table 4 further clarifies the mechanisms contributing to the enhanced performance of the CRJ specimen. In comparison to the PRJ specimen, the CRJ specimen exhibited an 81% enhancement in maximum lateral load and a 28% improvement in starting stiffness. The force associated with the onset of cracking rose by 28%, indicating enhanced elastic resistance and postponed crack initiation. The yielding force of the steel increased by 77%, with a 20% increase in the displacement at yielding. The strong correlation between the 77% increase in yielding force and the 81% increase in maximum force indicates that the primary factor responsible for the enhancement in peak strength originates from the improved flexural capacity and delayed yielding provided by the composite steel connection, rather than from an increase in stiffness alone. The enhanced initial stiffness contributes primarily to the elastic-stage response, whereas the composite connection facilitates improved load redistribution and reduces the stress concentration inside the joint core, thus permitting stable force development beyond yielding. The CRJ specimen exhibited enhanced strength, stiffness, and ductility under monotonic loading, confirming the efficacy of the composite-reinforced connection in improving the structural performance of precast beam–column joints.

### 4.2. Cyclic Loading Results

A cyclic loading test was conducted in which experimental observation and numerical simulation were used to evaluate the seismic behavior of the joint specimens, with skeleton curves as the primary findings. The numerical data enhanced the results by taking into account the shape of the damage to the concrete, yielding parameters, plastic strain equivalent metrics, stress patterns on the composite steel structure, and reaction force histories.

#### 4.2.1. CRJ Specimen

**Figure 14:**
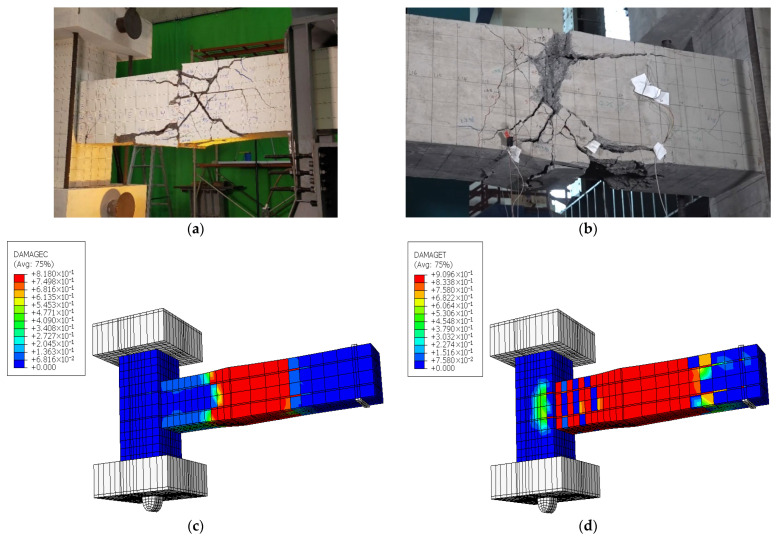
Comparisons of the deformed shape of the CRJ specimen: (**a**) experimental results, viewed from the left-hand side of the specimen; (**b**) experimental results, viewed from the right-hand side of the specimen; (**c**) compressive damage in the FE analysis; (**d**) tensile damage in the FE analysis. The color contours used for the numerical analysis results display the progression from a lower value (in blue color) to a higher value (in red color).

Figure 14 compares the deformed shapes and damage patterns for the CRJ specimen derived from experimental testing and numerical modeling. There is a robust correlation between the experimentally documented crack patterns depicted in Figure 14a,b and the numerical damage contours shown in Figure 14c,d, suggesting that the FE model effectively represents the overall deformation behavior and failure localization of the specimen. These findings indicate that the degradation and yielding of the concrete were primarily localized in the SRC-RC region of the beam, whereas the beam–column connection area remained essentially undamaged during the loading process. In the experimental specimen, diagonal and inclined fractures formed in the beam area near the composite connection, extending in the direction of the load. These areas of cracking are closely aligned with the regions of significant compressive and tensile damage predicted by the FE study, where damage values approached unity, indicating severe material deterioration.

The observed pattern of damage is attributed to the interaction between the embedded steel connection and the surrounding reinforced concrete. Following the application of cyclic loading, the steel structure directly interacted with the longitudinal reinforcement of the beam, resulting in concentrated cyclic lateral forces and elevated stress levels in both the upper and lower reinforcement layers. This interaction accelerated the yield of the beam reinforcement and promoted crack propagation in the SRC-RC region of the beam rather than within the joint core. The primary failure cracks can therefore be categorized as flexural-shear cracks, corresponding to a beam-controlled failure mechanism. Furthermore, the results from both the experimental observations and numerical studies indicate that plastic hinge formation occurred within the SRC-RC region rather than at the beam–column interface. The alignment of the plastic hinge positions and crack patterns between the two methods indicates significant agreement in the prediction of the deformed shapes. This conformity verifies that the numerical model accurately replicates the governing mechanisms of damage and validates its suitability for analyzing the cyclic behavior of the composite-reinforced joint.

However, although the FE model successfully captured the damage localization and plastic hinge formation, it failed to replicate the pinching behavior observed in the experimental setting. This limitation results from the implementation of perfect bond assumptions and continuum solid elements inside the CDP framework. In future work, the cyclic response prediction could be improved by integrating bond-slip interface models between the reinforcement and concrete, contact-based interactions between the steel connection components and adjacent concrete, or cohesive zone formulations to represent discrete crack progression. These improvements to the model would enable more precise simulation of stiffness deterioration and pinching phenomena during load reversal.

**Figure 15:**
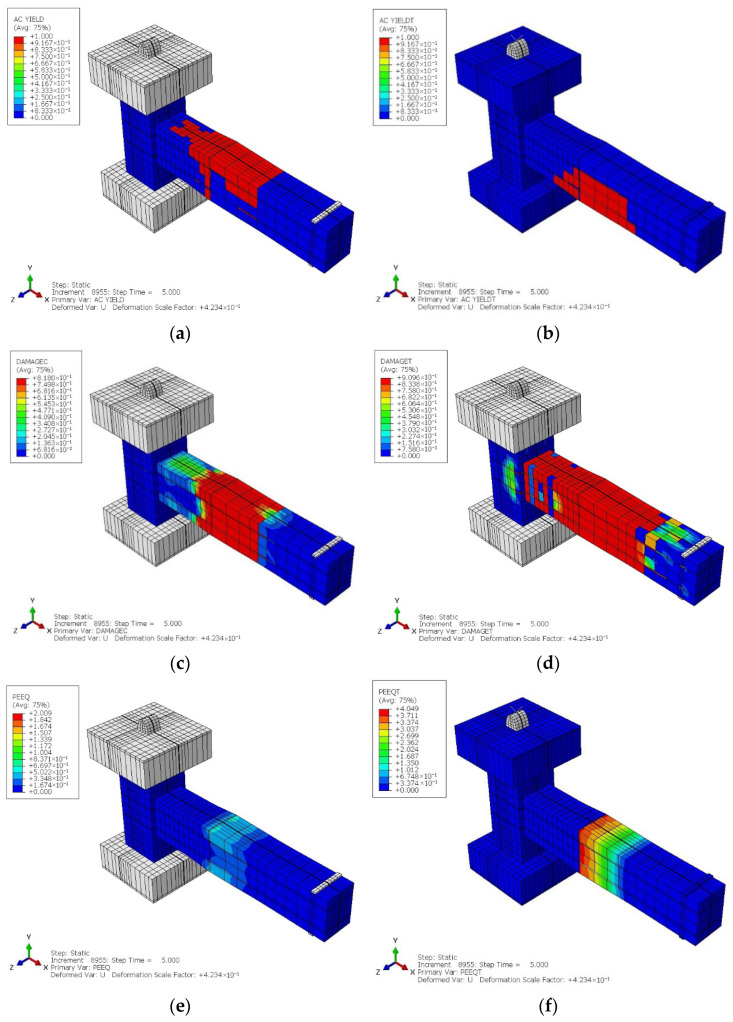
Results of a cyclic load FE analysis of the CRJ specimen, specifically for the concrete material: (**a**) yielding parameter in compression (AC YIELD value); (**b**) yielding parameter in tension (AC YIELDT value); (**c**) damage parameter in compression (DAMAGEC value); (**d**) damage parameter in tension (DAMAGET value); (**e**) plastic strain equivalent parameter in compression (PEEQ value); (**f**) plastic strain equivalent parameter in tension (PEEQT value). The color gradients illustrate the transition from a lower value (in a darker color) to a higher value (in a brighter color).

**Figure 16:**
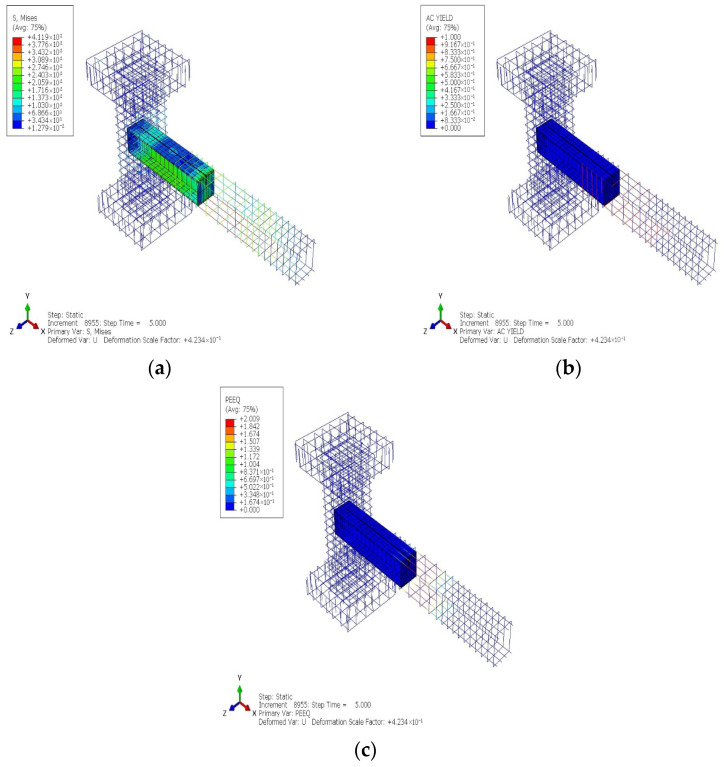
Results of a cyclic load FE analysis of the CRJ specimen, specifically for the steel components: (**a**) stress value; (**b**) yielding parameter in compression (AC YIELD value); (**c**) plastic strain equivalent parameter in compression (PEEQ value). The color gradients show the transition from a lower value (in a darker color) to a higher value (in a brighter color).

The cyclic analysis results for the CRJ specimen indicate a distinct response pattern. Figure 15a,b show the distribution of the yielding parameters, and it can be seen that the yielding condition occurred later and was more uniformly distributed, predominantly developing in the SRC-RC area rather than at the beam–column connection interface. This behavior indicates efficient load transmission and stress redistribution enabled by the composite steel component. From Figure 15c,d, which show the damage parameter contour distribution, it can be seen that concrete degradation was primarily localized in the beam region distant from the joint core, while the connection area remained predominantly intact during the loading history. Furthermore, the plastic strain equivalent parameter in compression (PEEQ) and plastic strain equivalent parameter in tension (PEEQT) results in Figure 15e,f indicate a gradual accumulation of plastic strain along the beam, corresponding to the development of a beam-controlled plastic hinge. Figure 16a–c show the distributions of steel stress, yielding metrics, and plastic strain, respectively. All of the contours confirm that the composite steel connection actively contributed to resisting cyclic demands, leading to a more stable and ductile deformation pattern under reversed loading.

#### 4.2.2. PRJ Specimen

**Figure 17:**
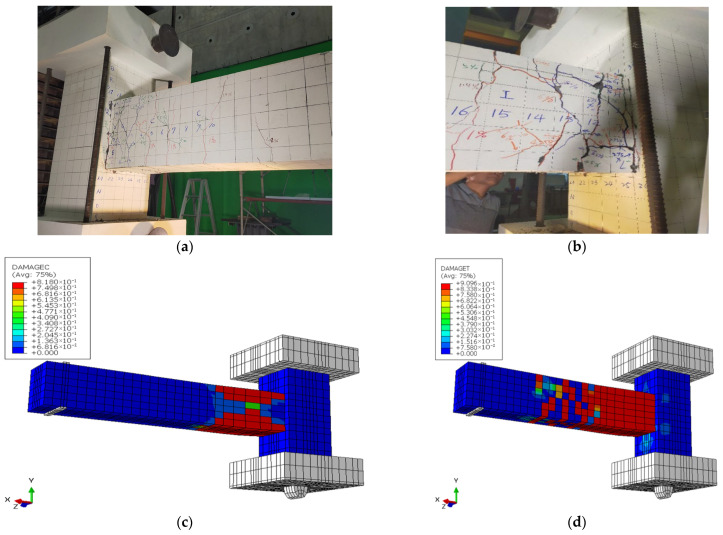
Comparison of the deformed shapes of the PRJ specimen: (**a**) experimental findings, observed from the left-hand side of the specimen; (**b**) experimental findings, observed from the right-hand side of the specimen; (**c**) compressive damage obtained from numerical analysis; (**d**) tensile damage obtained from numerical analysis. The color variations in the FE results represent the progression from a lower value (in blue color) to a higher value (in red color).

Figure 17 presents a comparison between the experimentally observed deformation patterns and the numerical damage contours of the PRJ specimen under cyclic loading. The experimental crack maps in Figure 17a,b reveal significant cracking centered at the beam–column interface, with cracks extending into the beam SC area. The damage pattern is reliably replicated in the FE findings, as evidenced by the compressive and tensile damage contours in Figure 17c,d. The numerical findings demonstrate that both compressive crushing and tensile cracking rapidly accumulated within the joint core, where significant stress demand developed during cyclic loading. The damage localization in this region indicates restricted redistribution of internal forces within the beam, resulting in gradual degradation of the connection. The color contours in the FE findings indicate elevated damage indices localized at the beam–column interface, a finding that corresponds closely with the experimentally observed fracture concentration and spalling in that area.

In contrast to the CRJ specimen, the PRJ specimen exhibited no significant displacement of damage from the joint core, nor was there any notable redistribution of plastic demand toward the beam region. The absence of a composite steel connection led to a restricted force transmission capacity and accelerated degradation within the joint core. The joint region governed the total response, indicating a joint-controlled failure mechanism. The similarity between the experimental crack patterns and numerical damage distributions indicates that the FE model accurately represents the mode of deformation and failure location of the PRJ specimen subjected to cyclic loading.

The progress of the stiffness degradation in the PRJ specimen was also analyzed by comparing the evolution of the secant stiffness obtained from the hysteresis envelopes. At lower drift ratios, the numerical model had a slightly higher initial stiffness relative to the experimental findings, which was principally attributed to the assumption of perfect bonding and the absence of bond-slip phenomena. As the drift ratio increased, both the numerical and experimental findings indicated a gradual drop in stiffness associated with crack propagation and damage accumulation within the joint core. There were similar patterns for the rate of degradation in the secant stiffness at moderate to large drift levels in the two methods, suggesting that although pinching behavior was not entirely captured numerically, the global degradation pattern influencing the structural response was adequately represented.

**Figure 18:**
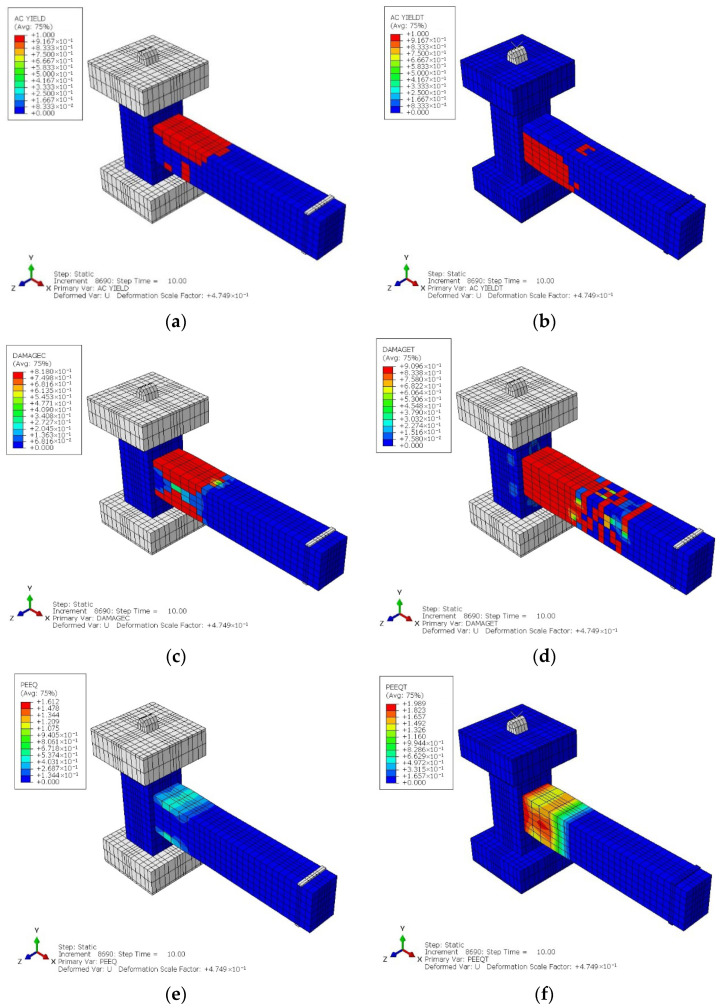
Results of a cyclic load FE analysis for the PRJ specimen, specifically for the concrete material: (**a**) yielding parameter in compression (AC YIELD value); (**b**) yielding parameter in tension (AC YIELDT value); (**c**) damage parameter in compression (DAMAGEC value); (**d**) damage parameter in tension (DAMAGET value); (**e**) plastic strain equivalent parameter in compression (PEEQ value); (**f**) plastic strain equivalent parameter in tension (PEEQT value). The color gradients illustrate the transition from a lower value (in blue color) to a higher value (in red color).

**Figure 19:**
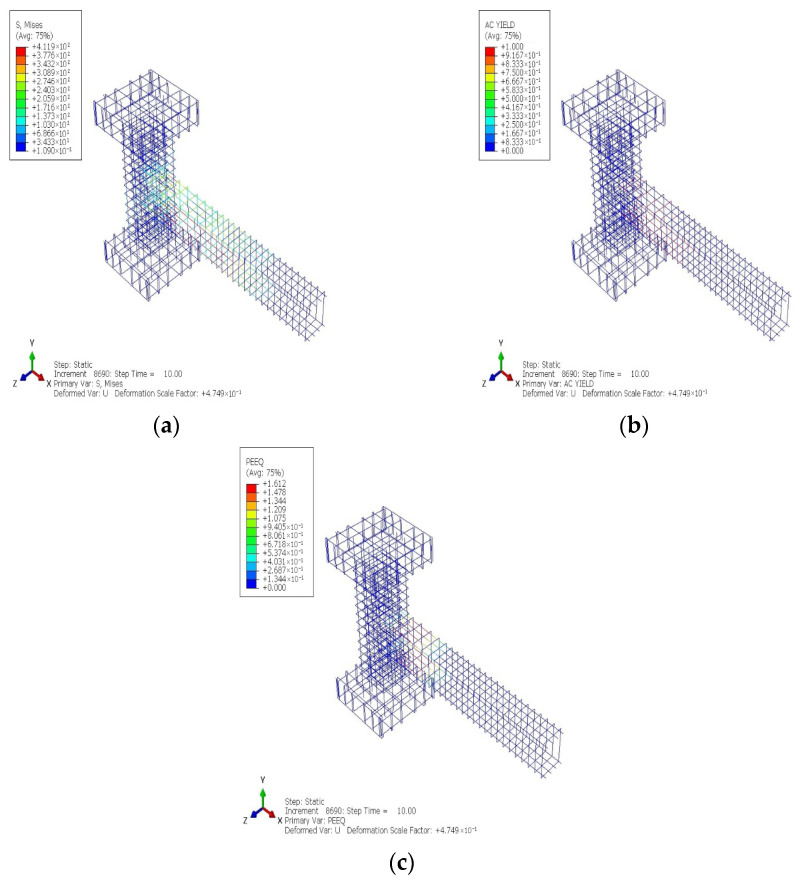
Results of a cyclic load FE analysis for the PRJ specimen, specifically for the steel components: (**a**) stress value; (**b**) yielding parameter in compression (AC YIELD value); (**c**) plastic strain equivalent parameter in compression (PEEQ value). The color gradients show the transition from a lower value (in blue color) to a higher value (in red color).

Figure 18 and Figure 19 show the cyclic analytical results for the PRJ specimen in terms of various parameters, which indicate that inelastic behavior was predominantly localized in the beam–column connection region. The AC YIELD and AC YIELDT contours shown in Figure 18a,b reveal the premature activation of yielding in the longitudinal reinforcement within the joint core, while the color variations indicate a restricted capacity for stress redistribution. Moreover, the DAMAGEC and DAMAGET data, shown in Figure 18c,d, suggest significant concrete degradation in both compression and tension at the connection and in the neighboring SC region, with damage values approaching the maximum limit at elevated drift levels. The values of PEEQ and PEEQT metrics in Figure 18e,f further confirm that the plastic strain rapidly became localized within the joint rather than spreading along the length of the beam. Meanwhile, the steel stress and strain contours in Figure 19a–c reveal a significant stress concentration at the beam–column interface, suggesting that the joint governed the cyclic response. These results provide evidence for a joint-controlled mechanism characterized by initial stiffness degradation, localized yielding, and restricted ductility during cyclic loading.

#### 4.2.3. Comparison of CRJ and PRJ Specimens

**Figure 20:**
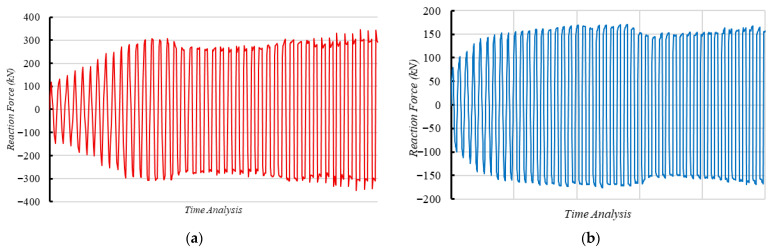
Results of an analysis of reaction force vs. time, derived from the FE simulation: (**a**) CRJ specimen model; (**b**) PRJ specimen model.

**Figure 21:**
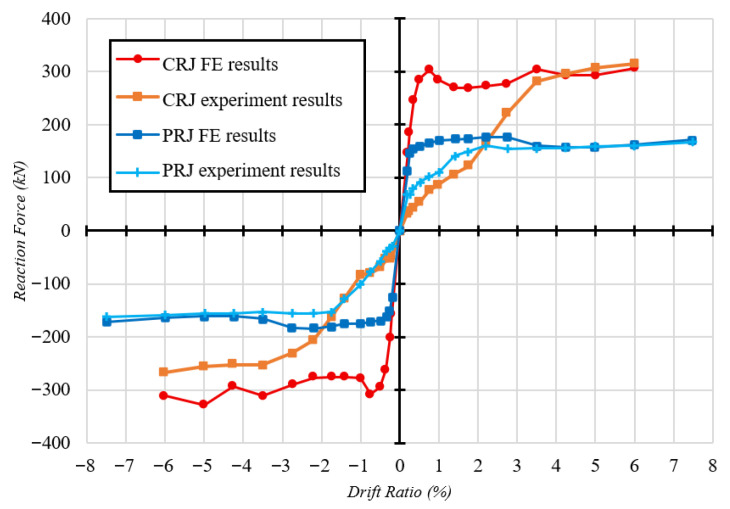
Skeleton curves for the CRJ and PRJ specimens derived from experimental and numerical findings.

Figure 20 presents the reaction force histories derived from the FE analysis for the CRJ and PRJ specimens subjected to cyclic loading. From Figure 20a, it can be seen that the CRJ specimen undergoes a gradual and stable increase in reaction force during the initial loading stages, indicating effective development of stiffness and strength. The reaction force rises until it reaches approximately 32% of the overall analysis length, after which a minor decline in peak force is observed. Despite this reduction, the specimen maintains a relatively consistent load-carrying capacity throughout subsequent cycles. This phenomenon is attributed to the strain-hardening characteristics of the steel components, which allow the composite connection to endure cyclic deformation without significant loss of strength. In contrast, the reaction force history for the PRJ specimen shown in Figure 20b consistently rises only during the initial phase of the loading history, reaching a stable response at roughly 10% of the overall analysis length. Beyond this point, the force amplitude remains nearly constant, indicating limited strain-hardening capacity and reduced ability to develop additional strength under increasing cyclic deformation. This reaction represents earlier stiffness degradation and a more brittle joint-controlled behavior compared with the composite-reinforced material. A quantitative comparison of the cyclic strength and degradation behavior is provided by the skeleton curves presented in Figure 21.

Figure 21 presents the skeleton curves derived from the cyclic hysteresis responses of both the CRJ and PRJ specimens, acquired from experimental testing and FE analysis. A comparison reveals major differences in strength development, deformation capacity, and the correlation between numerical and experimental results for the two joint configurations. Under cyclic loading, the CRJ specimen demonstrated significantly greater lateral resistance compared to the PRJ specimen, with maximum values roughly 92% higher than for the PRJ specimen for the experimental results and 93% higher for the numerical calculations. This significant improvement validates the efficacy of the composite steel connection in terms of improving joint strength and maintaining load-carrying capacity under elevated drift levels.

The skeleton curve for the CRJ specimen obtained from the FE model is closely aligned with the experimental ultimate forces for moderate to large drift ratios, particularly above a drift value of approximately 3.5%. At these deformation levels, both methods indicate stable elastic–plastic behavior with minimal strength deterioration. However, at lower drift ratios, the numerical results show a stiffer response and do not display the pinching behavior seen in the experiments. This limitation may lead to slight overestimates of the initial stiffness and energy dissipation at serviceability-level deformations; however, the numerical predictions are closely aligned with the experimental data at moderate to large drift ratios. Consequently, the evaluation of strength capacity, plastic hinge development, and design-level seismic performance remains unaffected.

The PRJ specimen exhibits a similar trend, although with reduced deformation capacity and strength. At small drift ratios, the agreement between numerical and experimental skeleton curves is constrained due to significant pinching and stiffness degradation in the experimental response. However, at drift values above about 2.2%, the FE predictions are closely aligned with the experimental peak forces in both loading directions. In this range, the numerical model accurately captures the post-yield strength progression of the PRJ specimen, while it fails to replicate the pinching effects evident in the laboratory testing.

A comparison of the skeleton curves demonstrates that the FE models can accurately anticipate the peak cyclic strength and post-yield behavior of both joint configurations at moderate to large drift ratios. While discrepancies at lower drift levels persist due to the assumption of perfect bonding and simplified crack representations, the numerical simulations successfully reproduce the relative performance variations between the CRJ and PRJ specimens, and particularly the significant strength and stability improvements afforded by the composite-reinforced joint configuration.

The energy dissipation capacity was assessed by calculating the cumulative area enclosed by the hysteresis loops derived from both the numerical simulations and experimental tests. The findings shown in Table 5 demonstrate that the CRJ specimen had consistently enhanced energy dissipation capacities relative to the PRJ specimen. The FE study revealed that the CRJ specimen dissipated a total energy of 1603.71 kJ at a drift ratio of 6%, while the PRJ specimen dissipated 950.69 kJ within the same drift range, corresponding to an increase of nearly 69%. The numerical results indicate that while the PRJ specimen had marginally greater energy dissipation at minimal drift ratios, the CRJ specimen outperformed the PRJ specimen when the drift ratio exceeded roughly 0.75%. Above this value, the energy dissipation per cycle for the CRJ specimen increased significantly, reflecting enhanced plastic deformation capacity and stable post-yield behavior facilitated by the composite steel connection. The experimental results indicated a more moderate improvement in energy dissipation: the cumulative energy dissipation of the CRJ specimens reached 458.87 kJ at a 6% drift ratio, in contrast to 357.98 kJ for the PRJ specimen, corresponding to an increase of roughly 28%. Although the total amount of dissipated energy varied from the computational predictions, the experimental patterns consistently validated the enhanced energy absorption capacity of the CRJ configuration.

The discrepancy between numerical and experimental energy dissipation is primarily attributed to the idealized modeling assumptions employed in the FE simulations. In the models, perfect bonding was assumed between the steel and concrete, and continuum solid elements were employed, which do not explicitly consider bond slip, crack opening, and closure behavior, and the cyclic degradation mechanisms that contribute to the pinching in the experimental findings. Consequently, the numerical simulations provided more stable elastic–plastic loops and overestimated the cumulative energy dissipation. In contrast, the experimental hysteresis responses demonstrated stiffness degradation and pinching effects linked to cyclic cracking, interface slip, and gradual damage accumulation. These mechanisms reduce the enclosed hysteresis area and hence the total energy dissipation. Despite these differences, both methodologies consistently show that the composite steel connection improves the energy dissipation capacity of the joint relative to the conventional joint configuration. From an engineering perspective, the experimentally measured energy dissipation should be considered as the realistic performance standard, whereas the numerical predictions represent an upper-bound estimate based on idealized modeling assumptions.

### 4.3. Moment Distribution

In 3D FE analysis based on solid continuum elements, internal bending moments are not directly available as standard historical outputs. In this study, we therefore employed manual computation using reaction forces and effective moment arms and extracted the sectional moment results via the free-body cut tool in ABAQUS software to evaluate the moment distribution.

**Figure 22:**
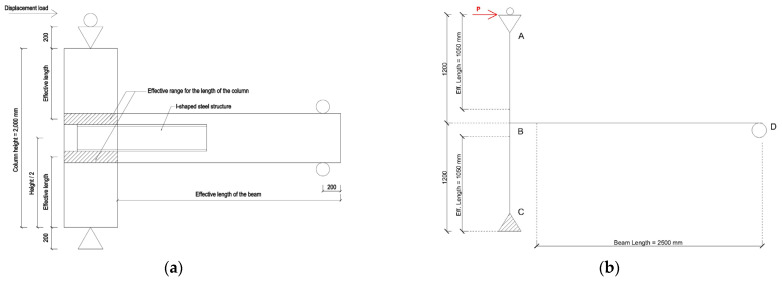
Simplified method for determining the effective length of beam–column joint specimens: (**a**) structural definitions; (**b**) one-dimensional model scheme of the structural specimens, where P denotes the reaction force, and A, C, B, and D represent the structural notations corresponding to the top and bottom supports of the column, the connection from the beam to the column, and the end-support of the beam, respectively.

The manual calculation method involved multiplying the lateral reaction force by the effective column length. Under the assumption of symmetry, the plastic moments of the upper and lower column segments were considered to be equal in magnitude but opposite in direction, while the plastic moment of the beam was assumed to be double that of the column, as shown in Equations (2)–(4), where *M_P_* represents the plastic moment from one point to another, as depicted in Figure 22. Furthermore, numerical analysis revealed that the maximum bending moment occurred at an effective column length (*L_eff_*) of approximately 925 to 1050 mm above the base, with a value of 1050 mm adopted for consistency. The plastic moment is linearly proportional to the effective length, meaning that the difference between 925 and 1050 mm yields an approximate difference of 12% in the calculated moment values. This sensitivity is considered acceptable for simplified analytical estimation and does not influence the identification of the governing plastic hinge location or the comprehensive interpretation of structural behavior. The simplified scheme for selecting the effective length for the moment calculation is illustrated in Figure 22.

*M_p.AB_* = *P* × *L_eff AB_* (2)

*M_p.BC_* = −*M_p.AB_* (3)

*M_p.BD_* = 2 × *M_p.AB_* = 2 × *P* × *L_eff AB_* (4)

The free-body cut function in ABAQUS was used to obtain moment values at specific sections through visualization of the resultant forces and the moment transmitted across the surfaces of the model [54]. However, the values of *M_p.AB_* and *M_p.BC_* derived with the free-body cut tool fluctuated when this strategy was employed. The concrete was represented using C3D8R elements, which allow for increased hourglass control in ABAQUS to mitigate zero-energy deformation modes. The numerical simulations demonstrated stable convergence and physically consistent deformation patterns, indicating excellent control of hourglass effects. The fluctuations in the moment values were therefore primarily attributed to stress redistribution and discretization effects, rather than solely to substantial hourglassing instability.

**Figure 23:**
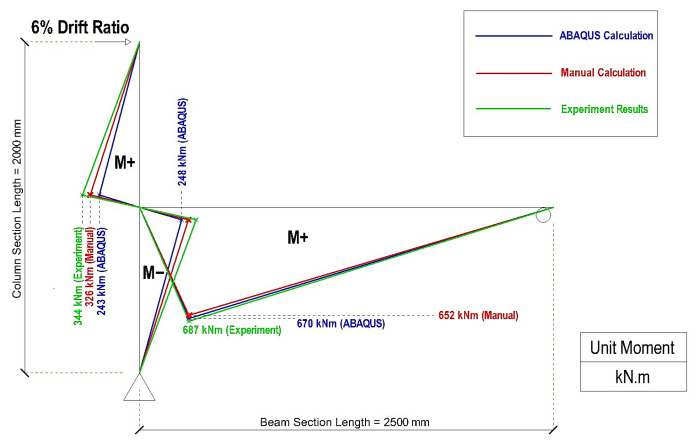
Plastic moment distribution for the CRJ specimen, derived from the results for the ultimate moment at a drift ratio of 6%.

**Figure 24:**
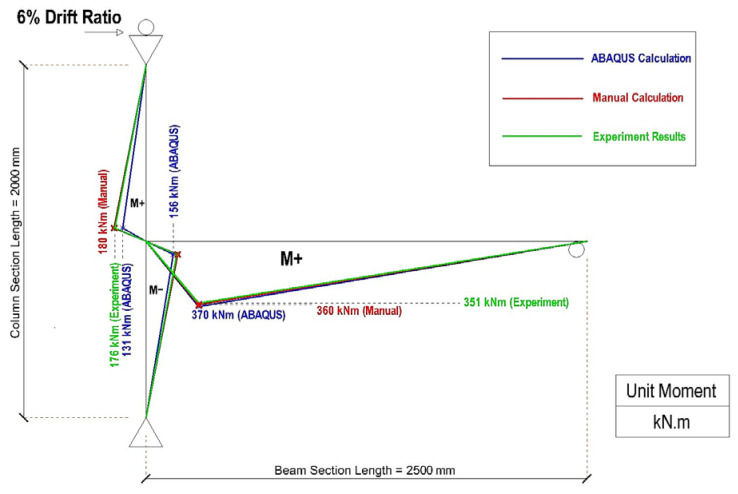
Plastic moment distribution for the PRJ specimen, derived from the results for the ultimate moment at a drift ratio of 7.5%.

Table 6 summarizes the results for the moment obtained from manual calculations based on experimental observation, numerical calculations, and the software, while Table 7 presents the values for the accuracy and the ratio of the moment value obtained with each type of moment calculation to the benchmark manual calculation based on the experimental results for the reaction force. Based on the data in Table 5 and Table 6, Figure 23 and Figure 24 depict the distribution of the plastic moment along the column and beam for the CRJ and PRJ specimens at their corresponding ultimate drift ratios. In both figures, the moment distributions derived from experimental results, manual computations, and ABAQUS simulations are superimposed to facilitate a direct comparison of the trends in magnitude and distribution.

The CRJ specimen depicted in Figure 23 exhibits a distinct concentration of plastic moment in the beam region, while the column moments are somewhat balanced and symmetrical. The maximum positive beam moment reaches approximately 687 kN·m in the experimental findings, a value that is closely aligned with the ABAQUS prediction of 657 kN·m and the value of 642 kN·m obtained from manual computation. This strong agreement verifies that the composite steel connection efficiently redirects the bending demand from the beam–column junction and facilitates beam-dominated plastic behavior. The column moments at sections AB and BC obtained with all three assessment methods exhibit consistent patterns, although slight variations are seen in the ABAQUS-derived values, especially around the column extremities. These differences are attributed to numerical issues linked to reduced-integration solid elements and the intrinsic challenge of deriving section moments from 3D continuum models.

Figure 24 shows the plastic moment distribution for the PRJ specimen, which exhibits a significantly decreased overall moment capacity compared to the CRJ specimen. The experimental value for the plastic moment is around 351 kN·m, in contrast to the values of 353 kN·m obtained from manual computation and 362 kN·m from ABAQUS simulation. Although the agreement in the beam moment is acceptable, significant disparities are seen for the column moments, particularly when using the free-body cut results from ABAQUS. This trend indicates that the inelastic demand is concentrated in the region of the beam–column joint, a finding that is aligned with the observed joint-dominated failure mode of the PRJ specimen.

A comparison of Figure 23 and Figure 24 shows that the composite-reinforced joint markedly improves the flexural capacity of the system and more evenly redistributes the internal forces. The beam plastic moment capacity for the CRJ specimen is around double that of the PRJ specimen, corresponding to the significant rise in ultimate lateral load observed in both the experimental and numerical findings. Furthermore, the more uniform moment gradient along the beam in the composite specimen signifies improved force transfer and reduced stress concentration at the joint core.

The results for the moment distribution indicate that the calculation methods derived from experimental reaction forces serve as the most dependable baseline for assessing plastic moments, although the software simulations reflect the overall moment distribution trend more accurately. Despite local inconsistencies, especially in the column moments, the computational results closely replicate the experimentally observed transition in plastic demand from the joint region to the beam region when a composite steel connection is used. These findings further substantiate the efficacy of the composite-reinforced joint in enhancing the strength capacity and facilitating a favorable ductile failure process.

## 5. Conclusions

This study presents a comprehensive experimental and numerical investigation of precast RC beam–column joints incorporating a composite steel connection, compared with a conventional pure reinforced joint. FE models were developed in ABAQUS software to simulate the experimental specimens and loading protocols, and nonlinear static monotonic and cyclic analyses were conducted under displacement-controlled loads. The computational framework was calibrated using experimentally obtained material properties and was validated through comparison of the load–displacement responses, damage patterns, deformed shapes, hysteresis behavior, energy dissipation, and moment distribution. Special attention was given to the failure mechanisms and plastic hinge development to enable an evaluation of the composite connection in controlling damage localization and improving seismic performance. The following conclusions can be drawn from these assessments:

- Nonlinear static analysis was suitable for simulating both monotonic and cyclic loading conditions, as the displacement-controlled loading protocol employed a sufficiently low loading rate, making inertia effects negligible. This method effectively captured the fundamental nonlinear response characteristics found in the experiments.
- In the CRJ specimen, premature degradation and the formation of plastic hinges in the area of the beam–column connection were successfully prevented. Inelastic deformation was intentionally directed to the SRC-RC region of the beam, whereas in the PRJ specimen, joint-controlled failure was observed, leading to significant concrete damage in the connection area.
- Under monotonic loading, the CRJ specimen exhibited significantly superior load-carrying capacity and initial stiffness compared to the PRJ specimen. With the composite connection, the initiation of cracking and steel yielding was delayed, and the post-yield deformation capacity was improved, indicating increased strength and stiffness arising from the embedded steel element.
- Under cyclic loading, the CRJ specimen exhibited prolonged stiffness and load resistance for a larger segment of the loading history, with a more stable hysteretic response. In contrast, the PRJ specimen showed premature stiffness degradation and reduction in strength, indicating its limited ability to withstand repeated inelastic deformation.
- The finite element models developed in ABAQUS accurately replicated the experimentally observed failure mechanisms and damage distributions, especially during the post-yield and strain-hardening phases. This finding confirms that the material constitutive models, element formulations, and boundary conditions chosen here accurately represented the overall structural responses of both joint configurations.
- Comparison of the skeleton curves revealed substantial agreement between calculated and experimental peak forces at moderate to large drift ratios for both specimens. The discrepancies at minimal drift levels were mostly attributed to the pinching behavior observed in the experiments, which was inadequately represented in the numerical simulations due to the assumption of perfect bonding and the use of continuum solid elements.
- The CRJ exhibited enhanced cyclic energy dissipation, with experimental findings revealing a 28% increase in cumulative energy absorption relative to the PRJ. Numerical studies predicted a higher improvement (69%), due to the idealized modeling assumptions that failed to account for pinching and bond-slip phenomena. Consequently, the experimental results are regarded as the primary indicator of realistic structural performance.
- The integration of a composite steel connection significantly improved the strength, stiffness, ductility, and damage resistance of the precast beam–column joints. The experimental and computational results indicate that this connection method offers a feasible and effective approach for enhancing the seismic performance and reliability of precast RC frame structures.

The conclusions of this study should be interpreted within the context of several limitations. In the experimental program, only a single specimen was tested for each configuration, and the results therefore provide a mechanistic understanding rather than a statistical generalization. The loading protocol was quasi-static and displacement-controlled, with no consideration of dynamic rate effects. In the numerical simulations, perfect bonding was assumed between the steel and concrete, the steel behavior was characterized as elastic–perfectly plastic without strain hardening, and the CDP parameters were adopted based on recognized values in the literature. These assumptions may have affected the predictions of the stiffness degradation and energy dissipation, especially under cyclic loading conditions.

At present, the proposed composite connection is applicable only to precast RC beam–column connections with similar geometric proportions, reinforcing configurations, axial load magnitudes, and quasi-static seismic loading conditions. Future investigations should include a variety of specimens, diverse axial load ratios, different steel section configurations, and enhanced bond-slip modeling to enable better validation and generalization of the suggested connection system.

In addition, the thermo-mechanical behavior of the proposed composite connection under elevated temperature conditions was not investigated. A previous study of RC members subjected to electric vehicle fire scenarios indicated that fire exposure can induce significant thermal gradients and accelerated degradation, especially in horizontal elements [55]. This current study promotes a beam-controlled inelastic mechanism based on the strong column–weak beam principle, necessitating an additional assessment of the efficacy of damage relocation under fire exposure, especially considering the embedded steel component and the potential for differential thermal expansion between steel and concrete. Future studies should therefore include coupled thermal-structural analysis and experimental validation to assess the residual strength, stiffness degradation, and modes of failure under combined seismic and fire loading scenarios.

## Figures and Tables

**Table 1:** Summary of precast concrete material properties.

Compressive Strength *f_c_′* (MPa)	Ultimate Strain *ε_u_* (%)	Modulus of Elasticity *E* (MPa)	Poisson’s Ratio υ	Density (kg/m^3^)
41.22	0.53	30,174.94	0.3	2400

**Table 2:** Geometry and reinforcement details of the CRJ and PRJ specimens.

Specimen	Structural Components	Cross-Sectional Dimensions (mm)	Length (mm)	Longitudinal Reinforcement	Stirrup Reinforcement	Tie Reinforcement
CRJ specimen	Column	600 × 600	2000	12#10	#4@100 mm	#4@100 mm
	Beam	450 × 550	2500	8#7	#4@100 mm	#4@100 mm
	Steel	304 × 301 × 11 × 17	1450	-	-	-
PRJ specimen	Column	600 × 600	2000	12#10	#4@100 mm	#4@100 mm
	Beam	450 × 550	2500	8#7	#4@100 mm	#4@100 mm

**Table 3:** Comparison of concrete damage responses using different solid element formulations implemented with the simplified cantilever column benchmark model.

Element Type	Formulation	Maximum Tensile Damage *d_t_* (%)	Maximum Compressive Damage *d_c_* (%)	Numerical Stability
C3D8I	Incompatible modes	135.8	178.0	Nonphysical damage/unstable
C3D8	Fully integrated	96.67	135.4	Nonphysical compressive damage
C3D8R	Reduced-integration	90.96	12.06	Stable
C3D8H	Hybrid formulation	96.37	11.59	Convergence instability
C3D8S	Improved surface stress	90.69	8.00	Stable
C3D8RH	Reduced, hybrid	90.69	9.09	Stable
C3D8IH	Incompatible, hybrid	125.5	13.78	Nonphysical damage
C3D8SH	Improved surface, hybrid	90.96	9.67	Stable

Note: Damage levels exceeding 100% (*d_t_* or *d_c_* > 1.0) indicate nonphysical damage progression inside the CDP framework and served as a criterion for the exclusion of inappropriate element formulations.

**Table 4:** Summary of results derived from the monotonic loading case.

Specimen	Max Force (kN)	*E* (MPa)	First Cracking		Yielding of Longitudinal Reinforcement	
			Force (kN)	Displacement (mm)	Force (kN)	Displacement (mm)
PRJ specimen	167.47	26,724	80.17	3	141.10	15
CRJ specimen	302.85	34,285	102.80	3	250.38	18
Percentage increase	81%	28%	28%	0%	77%	20%

**Table 5:** Summary of cumulative energy dissipation under cyclic loading up to 6% drift ratio.

Method	Specimen	Cumulative Energy Dissipation (kJ)	Increase Relative to PRJ Specimen
Numerical	PRJ	950.69	-
Numerical	CRJ	1603.71	+69%
Experimental	PRJ	357.98	-
Experimental	CRJ	458.87	+28%

**Table 6:** Summary of moment values obtained with the manual method and software approach.

Specimen	Method	Reaction Force (kN)	*M_p.AB_* (kN.m)	*M_p.BC_* (kN.m)	*M_p.BD_* (kN.m)
PRJ specimen	Manual (EXP) ^1^	167.09	176	176	351
	Manual (NUM) ^2^	167.815	177	177	353
	Software (FBC) ^3^	167.815	128	154	362
CRJ specimen	Manual (EXP) ^1^	327	344	344	687
	Manual (NUM) ^2^	305.695	321	321	642
	Software (FBC) ^3^	305.695	260	245	657

^1^ Calculation with the manual method based on the experimental results; ^2^ calculation with the manual method based on numerical results; ^3^ calculation with the software approach using the free-body cut tool in ABAQUS software (ver. 2017).

**Table 7:** Comparison of the ratio moment between the manual method based on the FE results and the ABAQUS approach relative to the experimental test results.

Method	PRJ Specimen			CRJ Specimen		
	*M_p.AB_*	*M_p.BC_*	*M_p.BD_*	*M_p.AB_*	*M_p.BC_*	*M_p.BD_*
Manual (NUM) ^1^	100.6%	100.6%	100.6%	93.3%	93.3%	93.4%
Software (FBC) ^2^	72.7%	87.5%	103.1%	75.6%	71.2%	95.6%

^1^ Calculation using the manual method based on the numerical results; ^2^ calculation using the software approach with the free-body cut tool in ABAQUS software.

## Data Availability

The original contributions presented in this study are included in the article. Further inquiries can be directed to the corresponding authors.

## Abbreviations

The following abbreviations are used in this manuscript:

RC	Reinforced Concrete
3D	Three-Dimensional
FE	Finite Element
ASTM	American Society for Testing and Materials
NCKU	National Cheng Kung University
ACI	American Concrete Institute
UTM	Universal Testing Machine
CRJ	Composite-Reinforced Joint
PRJ	Pure Reinforced Joint
C3D8	3D Eight-Node Solid Element
SRC	Steel-Reinforced Concrete
SC	Steel–Concrete
CDP	Concrete Damage Plasticity
FBC	Free-Body Cut
